# Metabolomics of the Antipyretic Effects of *Bubali Cornu* (Water Buffalo Horn) in Rats

**DOI:** 10.1371/journal.pone.0158478

**Published:** 2016-07-06

**Authors:** Rui Liu, Qiong Huang, Jinjun Shan, Jin-ao Duan, Zhenhua Zhu, Pei Liu, Yong Bian, Er-xin Shang, Dawei Qian

**Affiliations:** 1 Jiangsu Collaborative Innovation Center of Chinese Medicinal Resources Industrialization, and National and Local Collaborative Engineering Center of Chinese Medicinal Resources Industrialization and Formulae Innovative Medicine, Nanjing University of Chinese Medicine, Nanjing, China; 2 Jiangsu Key Laboratory of Research and Development in Marine Bio-resource Pharmaceutics, Nanjing University of Chinese Medicine, Nanjing, PR China; 3 Jiangsu Key Laboratory for High Technology Research of TCM Formulae, Nanjing University of Chinese Medicine, Nanjing, PR China; 4 The First Affiliated Hospital, Nanjing Medical University, Nanjing, PR China; 5 Jiangsu Key Laboratory of Pediatric Respiratory Disease, Institute of Pediatrics, Nanjing University of Chinese Medicine, Nanjing, China; University of Nebraska Medical Center, UNITED STATES

## Abstract

*Bubali Cornu* (water buffalo horn, WBH) has been used for thousands of years in traditional Chinese medicine (TCM) as an effective treatment for *heat*. In the present study, we have carried out a metabolomics profiling study on plasma and urine samples to explore potential biomarkers and determine how WBH exerts its antipyretic effects in yeast-induced pyrexia at a metabolomic level. Ultra-high performance liquid chromatography coupled with quadrupole time-of-flight tandem mass spectrometry (UPLC-Q-TOF-MS), together with multivariate statistical analysis, was used to detect and identify potential biomarkers associated with pyrexia and with WBH treatment. In total, sixteen endogenous metabolites were identified in plasma samples and twenty-one metabolites were detected in urine samples. The biomarkers identified in this study, using metabolic pathway analysis (MetPA), are involved in glycerophospholipid, arachidonic acid, amino acid, sphingolipid, and purine metabolism, all of which are disturbed in rats with pyrexia. As a result, WBH affect arachidonic acid metabolism and oxidative stress in yeast-induced pyrexia rats chiefly. The present study determines the important substances underlying the antipyretic efficacy of WBH at a metabolic level. It might pave the way for further investigations into the mechanisms of action of other animal horn-derived traditional Chinese medicines (TCMs).

## Introduction

*Rhinoceri Asiatici Cornu* (rhinoceros horn, RH) has been used in traditional Chinese medicine (TCM) for over 2000 years. RH is considered to be indispensable components of a number of remedies and, in recent decades, illegal hunting to satisfy increasing demand has led to drastic reductions in rhinoceros populations [1, 2]. The global rhinoceros population decreased from 70,000 to 11,000 between 1970 and 1987, a decrease of 85% [1]. Since the use of RH is now illegal or restricted, substitutes are needed to resolve the conflict between legislation and the apparent indispensability of RH in TCM and, importantly, substitutes should protect these highly endangered species [3]. It has been documented in *Ming Yi Bie Lu* around 220 AD that *Bubali Cornu* (water buffalo horn, WBH) can remove *toxic-heat*, reduce *heat* in the blood, and arrest convulsions [4]. WBH has similar inorganic and amino acid constituents, and comparable pharmacological properties compared with RH [5–9]. Because of its abundance, low price, and eutherapeutic effects, WBH has been used as a substitute for RH in the treatment of fever since 1970’s. Studies have shown that WBH can reduce the body temperature of animals with fever [1, 4, 10]. Our previous study showed that WBH extraction can decrease tumor necrosis factor-α (TNF-α)-induced prostaglandin E_2_ (PGE_2_) level in rat cerebral microvascular endothelial cells (rCMECs), and can protect rCMECs survival from hydrogen peroxide (H_2_O_2_)-induced toxicity by increasing superoxide dismutase (SOD) and catalase (CAT) enzyme activities [4].

Metabolomics can be used for the qualitative and quantitative analysis of small molecule metabolites present in biofluids, peripheral tissues, or the central nervous system, and shows great promise for identifying biomarkers of drug efficacy [11, 12]. Metabolomics adopts a ‘top-down’ strategy to study the function of an organism by investigating the end products of metabolic pathway and can be used to understand drug-target networks in a holistic context [13]. Metabolomics has become increasing important in a number of areas, including cancer, insomnia, diabetes, gut functional ecology, disease diagnosis, and dysmenorrhea syndrome, and has also been used for high throughput screening to investigate the antipyretic activity of TCM [14–17].

In the present investigation, we have developed a method based on metabolomics to identify potential biomarkers. Ultra-performance liquid chromatography electrospray ionization quadruple time-of-flight mass spectrometry (UPLC-Q-TOF-MS), together with multivariate statistical analysis, was used to evaluate pyrexia. Plasma and urine metabolic profiles were present by UPLC-Q-TOF-MS based metabolomic analysis, which used to investigate the effects of WBH intervention on fever rats. Metabolomic profiles of fever individuals treated with WBH were compared with fever individuals to find out and identify changed metabolites. Then some determined metabolites regulated significantly were further recognized as potential biomarkers. Finally, it can be explain how WBH exerts its antipyretic effect based on the metabolic relationships.

## Materials and Methods

### Chemicals and reagents

The WBH were purchased from the Wang’s slaughter house of Wharf town area of Huaian city (The Geographic coordinates was N 33°31′56.57″, E 118°55′41.45″), Jiangsu Province, China and authenticated by Prof. Dr. Jin-ao Duan. The horns were firstly cut into slices by slicing machine, the horn slices were dried for over 24 h in 50°C, and then dried slices were pulverized to give a fine powder at room temperature. Dried and powdered horn (500 g) was refluxed twice in water (5 L) for 8 h according to the method of Chinese Pharmacopoeia (2015 edition). The combined extracts were concentrated and lyophilized, about 18.5 g (yield 3.7%), then stored at –20°C. The lyophilized WBH sample was re-dissolved in 0.9% sterile saline at a concentration of 40 mg (lyophilized WBH sample)/mL as needed. The WBH sample was oral administered, the administered volume was 10 mL/kg for each rat.

Yeast was purchased from Anqi Co., Ltd. (Hubei, China). Glycoursodeoxycholic acid, deoxycholic acid, prostaglandin E_1_, aspirin and formic acid were obtained from Sigma—Aldrich Chemical Co. Ltd (St. Louis, MO, USA). Liquid chromatography (LC)-grade methanol and acetonitrile was purchased from Merck KGaA (Darmstadt, Germany). Ultra-high purity water was prepared using a Millipore-Q system (Millipore Corporation, Billerica, MA, USA).

### Ethics Statement

Water buffalo was sacrificed in strict accordance with the Slaughter Permission of the People's Government of Huaian. Permit number: Huaian [2008] No. 24. Water buffalo is a kind of economic animal in China, which do not involve endangered or protected species. All sampling procedures and experimental manipulations were approved by the Government of Wharf town of Huaian city.

Animal welfare and experimental procedures were carried out in strict accordance with the Regulations of Experiments Animal Administration issued by the State Committee of Science and Technology of the People’s Republic of China. Permit number: SCXK (SU) 2008–0004. This study was approved by Nanjing University of Chinese Medicine. The methods of euthanasia were also consistent with the recommendations of Regulations of Experiments Animal Administration of China. In the present study, rats were killed by an overdose of pentobarbital sodium.

### Animals

Male Sprague—Dawley rats (200 ± 20 g) were obtained from Shanghai SLAC Laboratory Animal Co., Ltd. (Shanghai, China). Animals were housed under a 12-hour light/dark cycle at a temperature of 22 ± 2°C and relative humidity of 55 ± 5%. The animals received a standard diet and water *ad libitum*.

### Effect of WBH on yeast-induced fever in rats

Initial rectal temperatures were recorded using a digital clinical thermometer (OMRON, Kizugawa City, Japan), inserted about 2 cm into the rectum. Rats with an initial rectal temperature of 37–38°C were selected for the study and randomly divided into four groups with eight animals in each group: A, normal group (no treatment, n = 8); B, fever group (subcutaneous injection of yeast, n = 8); C, WBH treated fever group (n = 8); and D, Aspirin treated fever group (n = 8). Pyrexia was induced by subcutaneous injection of 20% (w/v) yeast in 0.9% sterile saline (10 mL/kg), and then the health of rats was monitored every 30 min after yeast injection. Rectal temperatures were measured 6 h after the yeast injection and only rats with a temperature increased greater than 1.2°C were used in the study. After subcutaneous injection of yeast, the health of rats was monitored every 30 min and there was no rat showed ill health symptoms. 6 h after the yeast injection, rats were treated orally with WBH extract with a single dose (lyophilizate 400 mg/kg body weight, administered volume was 10 mL/kg). Rectal temperatures were recorded 0.5, 1, 1.5, 2 and 3 h after WBH treatment.

### Collection and preparation of plasma samples

Rats in each group were anesthetized with 10% chloral hydrate and blood samples were collected in heparinized Eppendorf tubes *via* the posterior venous plexus before administration of WBH and 1, 2, and 3 h after administration. Plasma samples for liquid chromatography coupled with mass spectrometry (LC-MS) analysis were obtained by protein precipitation using acetonitrile. Plasma samples (200 μL) were treated with acetonitrile (600 μL) and, after vortexing for 30 s and centrifugation at 15000×g for 10 min, the supernatant was evaporated to dryness under a gentle stream of nitrogen. The samples were reconstituted with mobile phase (200 μL) and aliquots (10 μL) were injected into the LC-MS system.

### Collection and preparation of urine samples

Rats were allowed to urinate naturally and urine was collected at the following time points: 12 h before subcutaneous injection of yeast, 0–3 h after injection of yeast, 3–6 h after injection of yeast, 6–9 h after injection of yeast (corresponding to 0–3h after oral administration of WBH), 9–12 h after injection of yeast (corresponding to 3–6 h after oral administration of WBH), 12–24 h after injection of yeast (corresponding to 6–18 h after oral administration of WBH). Urine was centrifuged at 15000×g for 10 min, and the supernatant was collected. Urine supernatant (400 μL) was added to acetonitrile (1200 μL) and the mixture vortex mixed for 30 s and then centrifuged at 10000 rpm for 10 min. The supernatant was evaporated to dryness under a gentle stream of nitrogen and reconstituted with mobile phase (200 μL) before injection of an aliquot (10 μL) into the LC-MS system.

### UPLC-Q TOF/MS conditions

The UPLC separations were performed using a Waters ACQUITY UPLC system (Waters Corporation, Milford, Massachusetts, USA). An Acquity UPLC BEH C_18_ column (2.1 mm × 100 mm, 1.7 μm) was used for all separations. The mobile phase was composed of (A) acetonitrile and (B) 0.1% (v/v) formic acid. In order to obtain good chromatographic profiles in UPLC system, different chromatographic methods were applied due to the existence of different polarity of compounds in plasma and urine. For separation of plasma samples, a linear gradient elution was used: 0–0.5 min, A: 5%; 0.5–10 min, A: 5%–100%; 10–12 min, A: 100%; 12–13 min, A: 100–5%. For separation of urine samples, a linear gradient elution was also used: 0–1 min, A: 2%; 1–6 min, A: 10%–20%; 6–8 min, A: 20%–40%; 8–12 min, A: 40%–80%; 12–13 min, A: 80–2%. The flow rate of the mobile phase in each case was 0.4 mL/min, and the column temperature was maintained at 35°C.

MS was performed using a Synapt Q-TOF instrument (Waters, Manchester, UK), operated using an electrospray ionization (ESI) source in positive and negative mode. The ionization source conditions were as follows: capillary voltage, 3.0 kV; source temperature, 120°C; and desolvation temperature, 350°C. The sampling cone voltage was set at 30 V, the extraction cone voltage was 2.0 V (for plasma samples) or 0.7 V (for urine samples), the trap collision energy was 6.0 V, the transfer collision energy was 4.0 V, the trap gas flow was 1.50 mL/min, and the ion energy was set at 1.0 V. Nitrogen and argon were used as cone and collision gases, respectively. The cone and desolvation gas flow rates were 50 and 600 L/h, respectively. A scan time of 0.5 s (for plasma samples) or 0.2 s (for urine samples) was used throughout, with an interval scan time of 0.02 s and a collision energy of 6 eV. The MS data were collected from m/z 100–1000 Da in positive and negative ion modes. Data acquisition and processing were performed using Masslynx V4.1 software (Waters Corporation, Milford, Massachusetts, USA).

To ensure accuracy during the MS analysis, Leucine-enkephalin was used as the lock mass, generating an [M + H]^+^ ion (m/z 556.2771) and [M−H]^−^ ion (m/z 554.2615) at a concentration of 200 pg/mL and flow rate of 100 μL/min. Dynamic range enhancement was applied throughout the MS experiment to ensure accurate mass measurement over a wider dynamic range.

### Data processing and multivariate analysis

Centroid and integrated raw mass spectrometric data for plasma and urine samples were processed using MassLynx V4.1 and MarkerLynx software (Waters Corporation). The intensity of each ion was normalized with respect to the total ion count to generate a data matrix that consisted of retention time, m/z value, and normalized peak area.

Multivariate statistical analysis in the form of principal components analysis (PCA) and orthogonal partial least-squared discriminant analysis (OPLS-DA) was carried out. SIMCA-P 12.0 software (Umetrics AB, Umeå, Sweden) was used for the chemometric analysis. The quality of the model was described by the cross-validation parameter *Q*^2^, indicating the predictability of the model, and *R*^*2*^*Y*, which represents the total explained variation for the *X* matrix. S-plot and variable importance in the projection (VIP) were used for the selection of potential biomarkers. In the present study, variables with VIP values > 2 were considered to better explain *Y* (response).

### Biomarker identification and metabolic pathway analysis

To identify biomarkers, the accurate mass and fragment information obtained from UPLC-Q-TOF MS in positive and negative ion modes was compared with data published in the Human Metabolome Database (HMDB) (http://www.hmdb.ca/), the Kyoto Encyclopedia of Genes and Genomes (KEGG) (http://www.genome.jp/kegg/), the METLIN metabolite database (http://metlin.scripps.edu/) and Chemspider (http://www.chemspider.com).

Metabolic pathway analysis (MetPA) to identify and visualize affected metabolic pathways was carried out using a web-based tool (http://www.metaboanalyst.ca/faces/Secure/upload/PathUploadView.xhtml), based on sources including KEGG, the Small Molecule Pathway Database (SMPD) (http://www.smpdb.ca/), and the METLIN metabolite database.

## Results

### Rat rectal temperature measurements

**Fig 1:**
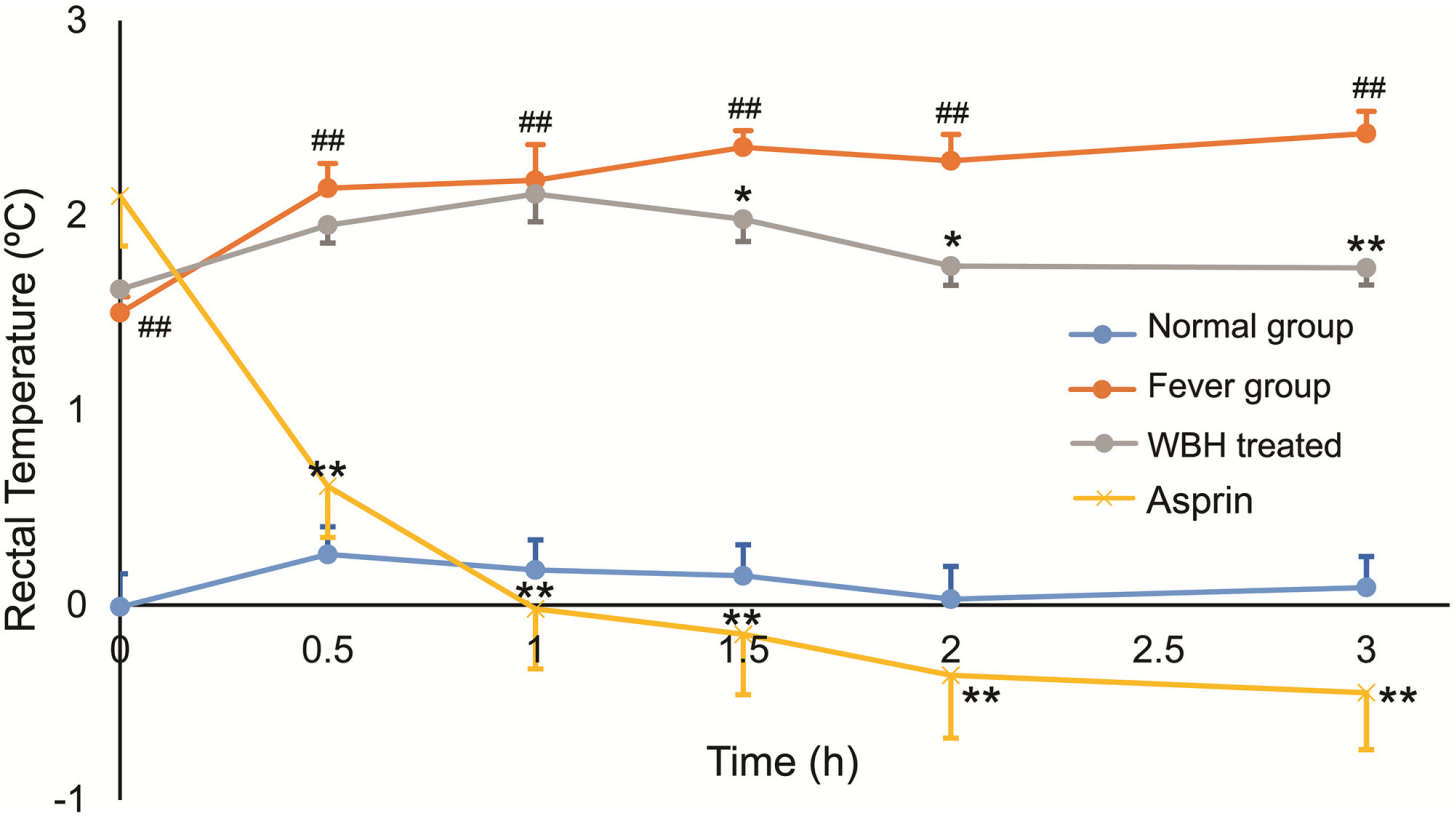
The increase of rats’ rectal temperatures. Each point represents the mean ± SEM, n = 8. #*p* < 0.05, ##*p* < 0.01 compared to the normal group; *p < 0.05, **p < 0.01 compared to the fever group.

Rectal temperatures of the rats at different time points are shown in Fig 1. When compared with the fever group, rectal temperatures increased significantly 6 h after subcutaneous injection of yeast (*p* < 0.01). Treatment with WBH extract significantly attenuated the yeast-induced increase in body temperature (*p* < 0.01, *p* < 0.05); the onset of action was 1.5 h after administration and the effect was maintained for 1.5 h. Aspirin could decrease the body temperature obviously (*p* < 0.01, *p* < 0.05)

### Multivariate data analysis of UPLC-ESI-Q-TOF MS output

**Fig 2:**
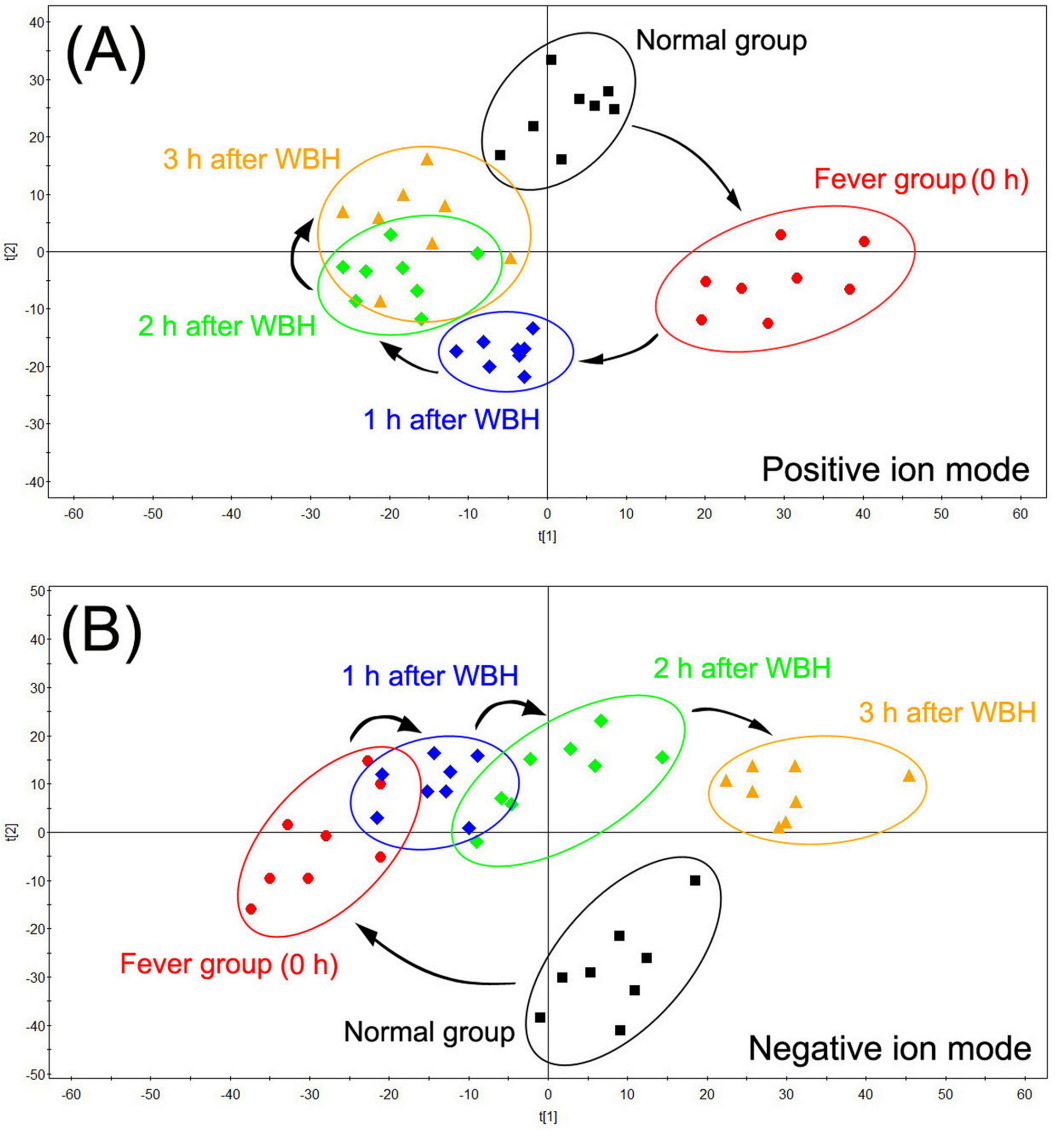
PCA analytical results of plasma from hyperthermia rats treated with WBH in different time period in positive mode (A) and in negative mode (B).

**Fig 3:**
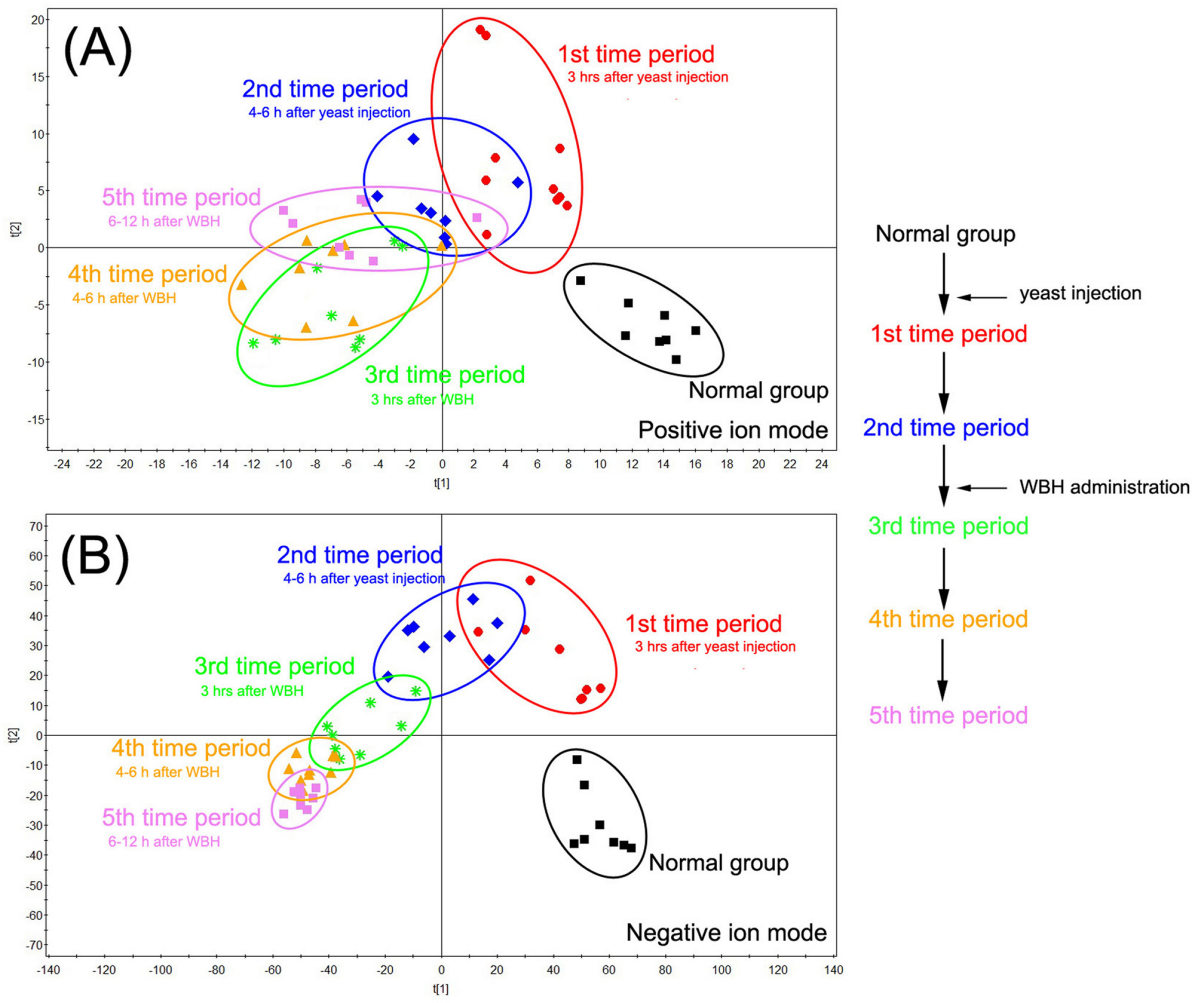
PCA analytical of urine results from hyperthermia rats treated with WBH in different time period in positive mode (A) and in negative mode (B).

UPLC-Q-TOF MS base peak intensity (BPI) chromatograms of plasma and urine samples are shown in Figures A and B in S1 File. An unsupervised PCA model was used to separate plasma and urine samples into two clusters for rats with fever (fever group) and normal (normal group) rats (Figures C and D in S1 File). Pyrexia was found to cause a disorder of endogenous metabolites in rats and time-dependent changes in plasma metabolites are shown in Fig 2. Six hours after yeast injection, plasma samples from the normal group and fever group were separated into two blocks, indicating a significant difference in the rats with pyrexia. Three hours after treatment with WBH extract, parameters for the rats with fever tend to resemble those of normal rats (normal group). The time-dependent changes in urine samples are shown in Fig 3, the tendency of WBH treated fever group resemble to normal rats was not significant. While it was obviously that 12 h after WBH treated, fever group far away from its initial position.

Based on these results, a supervised OPLS-DA was implemented to search for distinct metabolites between the normal group and fever groups. The parameters *R*^*2*^*Y* and *Q*^*2*^ for plasma and urine samples, which indicate that the established model has strong predictability, are shown in Table 1. The OPLS-DA S-plots are shown in Figure C (C) and (D) in S1 File; components located at the two ends of “S” might contribute significantly to the clustering of the normal group and the fever group. Components were extracted as potential biomarkers when their VIP values were >2. An independent *t*-test was performed, and those metabolites with significant differences between the normal group and fever groups (*p* < 0.05 or *p* < 0.01) were regarded to be biomarkers of pyrexia in rats.

**Table 1:** The parameters of *R*^*2*^*Y and Q*^*2*^ of plasma, urine and hypothalamus.

	Plasma		Urine	
	positive	negative	positive	negative
*R*^*2*^*Y*	0.999	0.991	0.986	0.989
*Q*^*2*^	0.885	0.868	0.974	0.959

### Identification of potential biomarkers

Biomarkers were tentatively identified by comparing the accurate mass and MS^E^ fragment information with data in the biochemical databases. Precise molecular masses were determined within measurement errors (<5 ppm) by Q-TOF MS, allowing the potential elemental composition, degree of unsaturation and fractional isotope abundance of the compounds to be determined. The presumed molecular formulae were then compared with data in the METLIN metabolite database and HMDB, leading to the identification of 16 endogenous metabolites in plasma and 21 metabolites in urine (S1 Table).

Compared with the normal group, LysoPE(22:0/0:0), LysoPC(20:4(5Z,8Z,11Z,14Z)), LysoPC(20:3(5Z,8Z,11Z)), LysoPC(20:1(11Z)), LysoPC(22:6(4Z,7Z,10Z,13Z,16Z,19Z)), LysoPC(16:1(9Z)), deoxycholic acid, glycoursodeoxycholic acid, glycocholic acid and LysoPE(0:0/22:5(7Z,10Z,13Z,16Z,19Z)) were significantly down-regulated (*p* < 0.05, or *p* < 0.01) in plasma from rats injected with yeast. Prostaglandin J_2_, hexadecanedioic acid, leukotriene A_4_, LysoPE(18:0/0:0), leukotriene D_5_, and LysoPC(22:5(4Z,7Z,10Z,13Z,16Z)) (*p* < 0.05, or *p* < 0.01) were significantly up-regulated compared with the negative control group.

In urine, dihydroceramide, dodecanedioic acid, uracil, homoanserine, xanthine, prostaglandin E_1_, aminoadipic acid, palmitoylglycine, norepinephrine sulfate, deoxyadenosine, tyramine, urocanic acid, xanthosine, *N*-acetylglutamine, L-aspartyl-4-phosphate, and 17-hydroxyprogesterone were significantly up-regulated (*p* < 0.05, or *p* < 0.01) whereas homoanserine, 4-hydroxynonenal, cystine, 2-pyrocatechuic acid, aspartyl-tyrosine, and tyrosyl-glutamine were noticeably down-regulated (*p* < 0.05, or *p* < 0.01).

**Fig 4:**
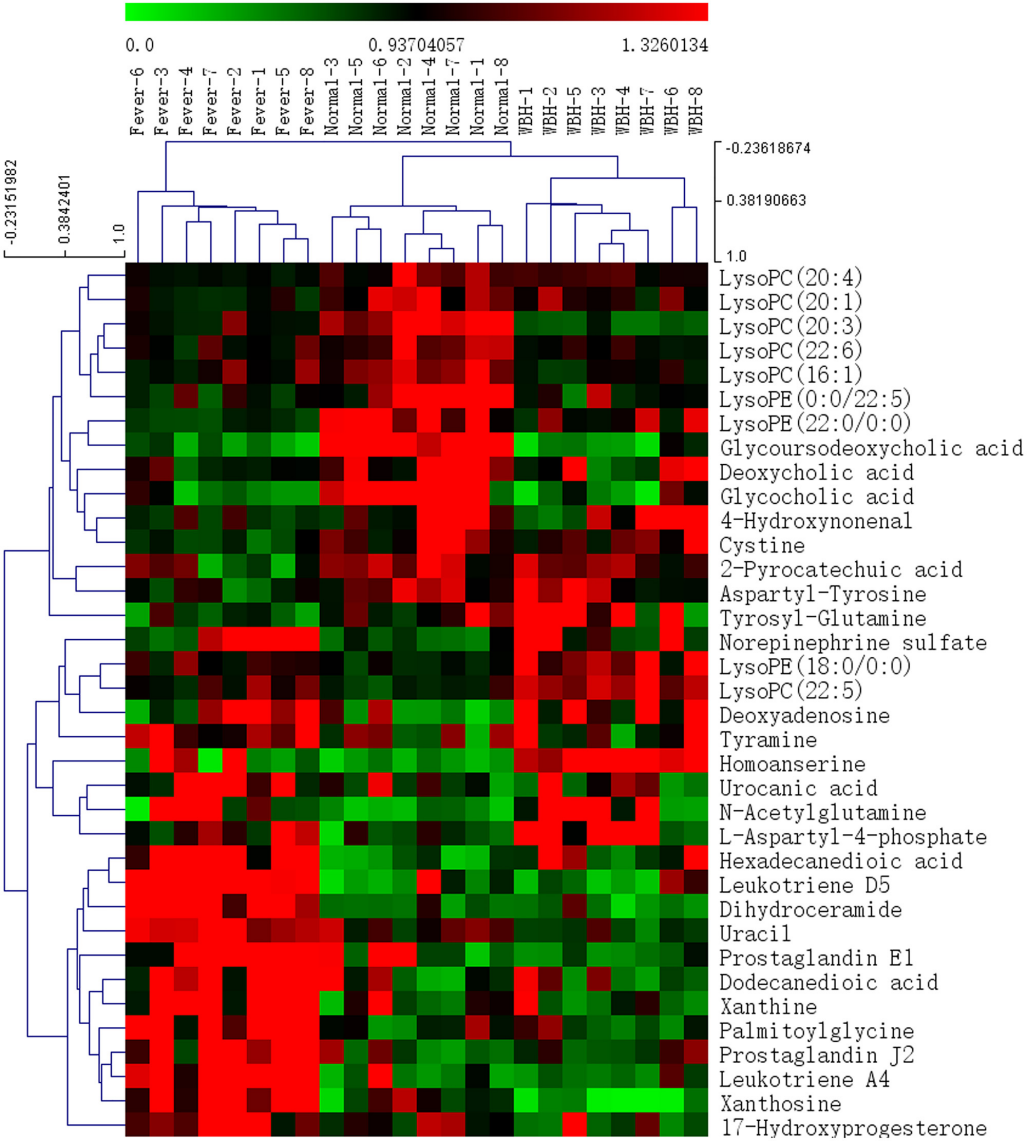
Heat maps from UPLC-MS. Fingerprinting of endogenous metabolites from the Normal group, Fever group and WBH treated group. Green represents negative values, red represents positive values.

The heat map (Fig 4, S1 Table) showed that the levels of metabolites dihydroceramide, dodecanedioic acid, uracil, xanthine, prostaglandin E_1_, palmitoylglycine, prostaglandin J_2_, hexadecanedioic acid, leukotriene A_4_, leukotriene D_5_, xanthosine, and 17-hydroxyprogesterone were up-regulated (*p* < 0.05, or *p* < 0.01) in fever rats and further down-regulated significantly (*p* < 0.05, or *p* < 0.01) after WBH treatment. Levels of LysoPE(22:0/0:0), LysoPC(20:4), LysoPC(20:1), cystine, and tyrosyl-glutamine were down-regulated (*p* < 0.05, or *p* < 0.01) in fever rats and then up-regulated significantly (*p* < 0.05, or *p* < 0.01) after WBH treatment. It was suggest that WBH can effectively regulate metabolic pathway associated with some metabolites produced in rats after yeast-induced pyrexia.

### Metabolic pathway and function analysis

**Fig 5:**
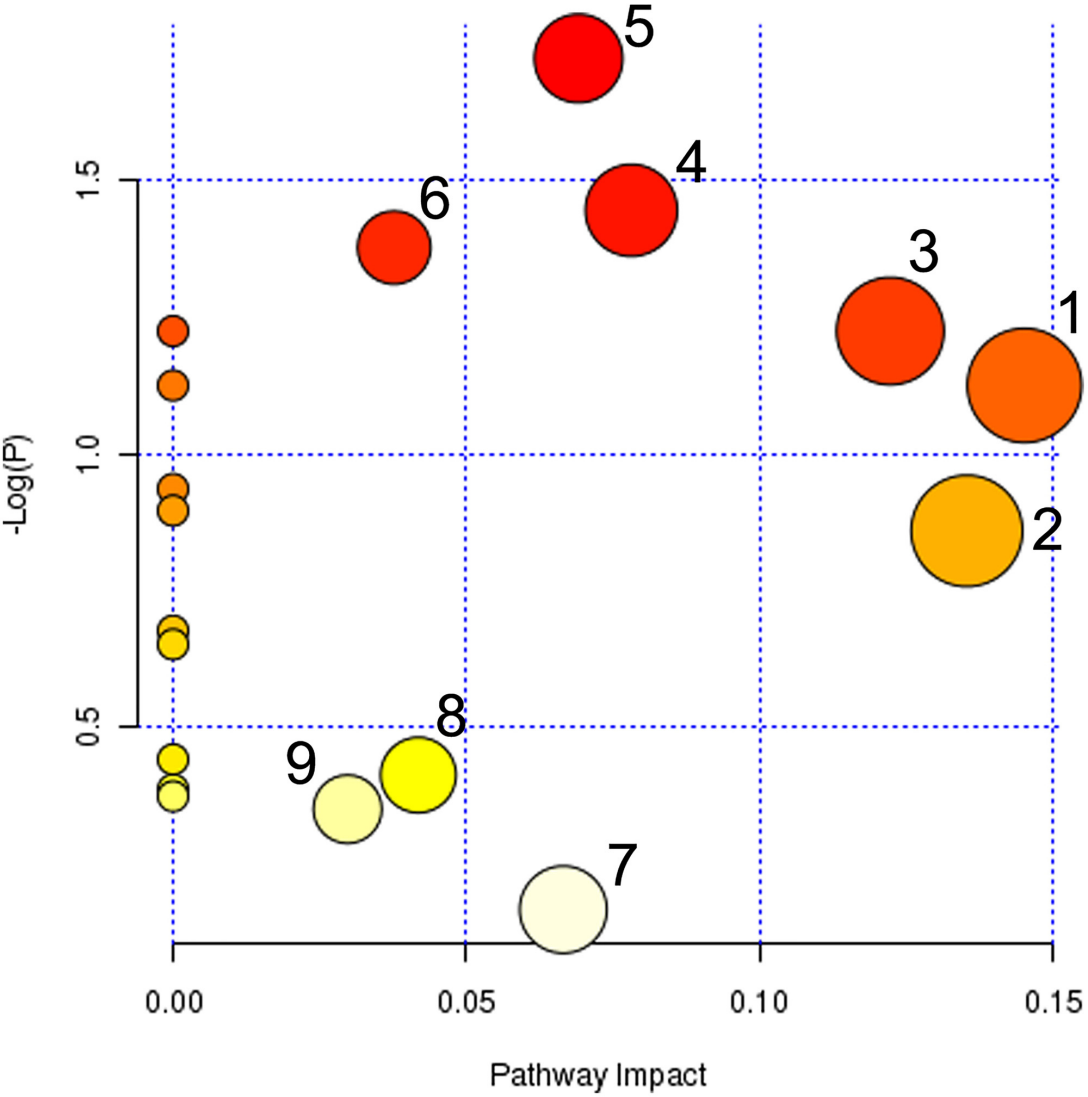
Summary of pathway analysis with MetPA. (1) Histidine metabolism; (2) Sphingolipid metabolism; (3) Tyrosine metabolism; (4) Arachidonic acid metabolism; (5) Glycerophospholipid metabolism; (6) Purine metabolism; (7) Steroid hormone biosynthesis; (8) Pyrimidine metabolism; (9) Primary bile acid biosynthesis.

Metabolic pathway analysis revealed that the biomarkers identified in plasma and urine play important roles in fever. As shown in Fig 5, the selected metabolites are involved in histidine metabolism, sphingolipid metabolism, tyrosine metabolism, arachidonic acid metabolism, glycerophospholipid metabolism, purine metabolism, steroid hormone biosynthesis, and pyrimidine metabolism. We suggest that these pathways may be modified by treatment with WBH.

## Discussion

**Fig 6:**
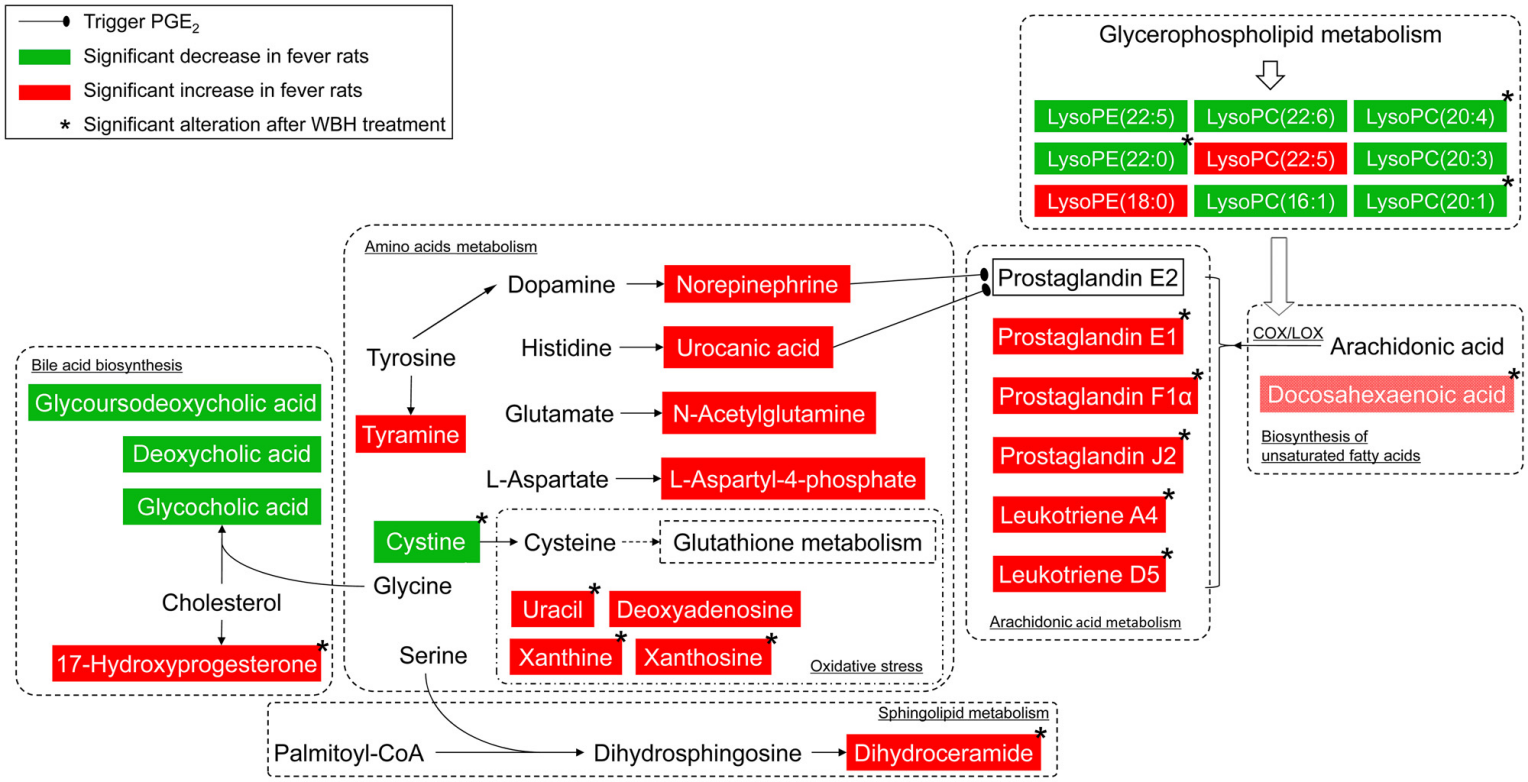
The metabolic network profile based on the known metabolic pathways.

In the present study, the antipyretic effect of WBH extract was evaluated using a metabolomic study based on UPLC-Q-TOF-MS. Yeast-induced fever is a classical model for pharmacological studies on the antipyretic effect of different compounds and preparations. Metabolic profiling studies of plasma and urine samples using UPLC-Q-TOF-MS, and gas chromatography coupled with mass spectrometer (GC-MS) combined with nuclear magnetic resonance (NMR) spectroscopy found that amino acid metabolism, together with energy, lipid and glycol metabolism, was involved in yeast-induced fever [14, 15, 18]. In the present study, we observed that WBH significantly reduced the temperature of rats with pyrexia. It was proposed that the fever model involve metabolic pathways including arachidonic acid, sphingolipid, glycerophospholipid, amino acid metabolism, etc. The antipyretic effects of WBH could involve metabolic pathways mainly including arachidonic acid, amino acid metabolism, and oxidative stress. The relationship pathway of these metabolites was mapped according to current knowledge of metabolic pathways (Fig 6).

Previous investigations have shown that aqueous extracts of WBH can significantly reduce the body temperature of febrile animals, in models of both infectious fever and noninfectious fever [4]. Another animal horns, such as SAH and goat horn, have been shown to significantly reduce the temperature of febrile rabbits and also to reduce levels of prostaglandin E_1_ (PGE_1_), PGE_2_, and tumor necrosis factor-α (TNF-α) in the serum [19]. In arachidonic acid metabolism, arachidonic acid can be stereospecifically oxygenated by cyclooxygenase-2 (COX-2) to prostaglandins (PGs) or by lipoxygenase (LOX) to leukotrienes (LTs) [20]. The inflammatory response to PGs and LTs is mediated through specific G protein-coupled receptors [21, 22]. PGs are important mediators of pyrexia, especially PGE_2_ can up-regulate the temperature set point of the hypothalamus preoptic area in the anterior hypothalamus (POAH) and trigger fever. In the present study, levels of PGs and LTs in plasma increased noticeably in rats showing fever. After treatment with WBH, PGs and LTs were significantly down-regulated, suggesting that WBH might exert its antipyretic effect by affecting PG-related pyrexia.

Lysophospholipids (LPCs), including LysoPE(22:0/0:0), LysoPC(20:4(5Z,8Z,11Z,14Z)), LysoPC(20:3(5Z,8Z,11Z)), LysoPC(20:1(11Z)), LysoPC(22:6(4Z,7Z,10Z,13Z,16Z,19Z)), LysoPC(16:1(9Z)), LysoPE(18:0/0:0), LysoPE(0:0/22:5(7Z,10Z,13Z,16Z,19Z)), and LysoPC(22:5(4Z,7Z,10Z,13Z,16Z)), are metabolites of glycerophospholipids that are formed by hydrolysis of phosphatidylcholine by the enzyme phospholipase A_2_ (PLA_2_). The degradation of glycerophospholipids by PLA_2_ also produces arachidonic acid, which then undergoes further metabolism. Although the exact relationship between LPCs and fever remains unclear, LPCs of different length and degree of saturation would have different capacities for inducing COX-2 expression during fever, for example, LPC 16:0 and 20:4 could promote both COX-2 expression and its mRNA synthesis [23]. It has been demonstrated that saturated LPCs increase due to the oxidation of unsaturated LPCs by reactive oxygen species (ROS) under inflammatory conditions [24]. The saturated LPCs could induce inflammation *in vivo*, and then the inflammation could be attenuated by unsaturated LPCs [25, 26]. In the present study, the amounts of LPE 22:0, LPE 22:5, LPC 20:4, LPC 20:3, LPC 20:1, LPC 22:6, and LPC 16:1 were decreased significantly, while the amounts of LPC 22:5 and LPE 18:0 were increased. Most unsaturated LPCs were down-regulated in the plasma of febrile rats. It was suggest that the regulation of LPCs in the present study showed little relationship with WBH treatment. Nevertheless, it is interesting that LPC 20:4, 20:1 and LPE 22:0 were significantly increased after WBH treatment. Therefore, it can be elucidated that WBH might not exert its antipyretic activity by interfering with the LPC metabolic pathway.

Norepinephrine sulfate (NES), tyramine, urocanic acid, cysteine, *N*-acetylglutamine, homoanserine, aminoadipic acid, and L-aspartyl-4-phosphate are formed during amino acid metabolism. NE is the principal transmitter of most postganglionic sympathetic fibers, and can be produced from dopamine by dopamine β-monooxygenase. In rats with lipopolysaccharide (LPS)-induced pyrexia, biosynthesis of NE was stimulated and microdialyzing NE was shown to raise core temperature [27, 28]. Both *in vivo* and *in vitro* investigations have shown that NE can trigger the release of PGE_2_ [27]. It has also been reported that a major part of NE is sulfoconjugated in blood or at sites easily accessible from blood [29]. The catecholamine-releasing compound tyramine, which is derived from tyrosine and acts as a neurotransmitter *via* a G protein-coupled receptor, is metabolized by the enzyme monoamine oxidase. In the present study, levels of NES and tyramine were increased in rats with fever. It has been demonstrated that catecholamines play an important role in the febrile response. Administration of WBH did not noticeably affect NES and tyramine levels.

Oral administration of WBH decreased levels of urocanic acid and increased levels of cystine. Urocanic acid is formed from L-histidine through the action of histidine ammonia lyase. It has been shown that treatment of normal human epidermal keratinocytes with *cis*-urocanic acid results in increased synthesis of PGE_2_ and cell death [30]. Cysteine is an important antioxidant that is unique amongst the twenty natural amino acids as it contains a thiol group, which can undergo oxidation/reduction (redox) reactions. Cysteine is one of the amino acids that make up the antioxidant tripeptide glutathione, and is often involved in electron-transfer reactions. Fever causes disordered metabolism and results in the generation of excessive free radicals; as a natural antioxidant, cysteine can act as a radical scavenger. It has been reported that *N*-acetylcysteine attenuated yeast-induced fever, and decreased IL-1β and TNF-α levels [31]. In the present study, levels of urocanic acid were increased and levels of cysteine significantly decreased in the febrile rats. Treatment with WBH did not significantly alter levels of homoanserine, aminoadipic acid, or L-aspartyl-4-phosphate. WBH may thus contribute to the relief of fever by modifying the metabolism of urocanic acid and cysteine.

Deoxycholic acid, glycoursodeoxycholic acid, and glycocholic acid, which are steroidal amphipathic molecules derived from the catabolism of cholesterol, were found in the plasma of rats with fever. A decrease in serum cholesterol levels has been shown to be a prognostic marker in neutropenic patients with fever [32]. A decrement in total cholesterol was observed during pyrexia in children and these changes were associated with the duration of the fever; the longer the fever, the more intense the alterations [33]. In the present study, cholic acids were decreased by yeast-induced pyrexia and treatment with WBH did not significantly alter levels.

As a COX-2 inhibitor, aspirin possess good antipyretic and anti-inflammatory effects. In the present study, metabolic profiles of WBH treatment and aspirin treatment were compared. As shown in Figures E, F, and I in S1 File, and S2 Table, both WBH and aspirin could reduce PGE_1_ level. Aspirin could increase the levels of glycoursodeoxycholic acid, glycocholic acid, and coprocholic acid, and increase the level of Glucose 1-phosphate, while WBH could not. Besides, WBH could alter the level of L-Cysteine and phytosphingosine, and aspirin could not. Based on these results, it might be the different type of antipyretic effects of WBH and aspirin. Aspirin might exert its antipyretic effect by taking part in bile acid biosynthesis, glycerophospholipid metabolism, arachidonic acid metabolism and some amino acid metabolism, yet WBH might by taking part in cysteine and methionine metabolism and arachidonic acid metabolism.

In summary, it can be indicated that WBH exert its antipyretic effect by affecting arachidonic acid and oxidative stress chiefly, including PGs, LTs, cystine, etc. It was consistent with our previous investigation [4]. Other metabolisms, such as bile acid biosynthesis, amino acids metabolism, glycerophospholipid metabolism and sphingolipid metabolism might be little interfered by WBH treatment. Several other metabolites, including dihydroceramide, docosahexaenoic acid, and 17-hydroxyprogesterone were also found to be involved in the pathology of fever and their levels altered significantly after treatment with WBH.

## Conclusion

After administration of WBH, the endogenous metabolites profile was significantly different compared with that of untreated febrile rats. The levels of a number of PGs, LTs, amino acids, sphingolipids, and purines showed significant differences before and after treatment with WBH. Arachidonic acid and oxidative stress were mainly involved in the antipyretic efficacy of WBH. The present study provides an insight into yeast-induced changes in metabolism in plasma and urine, and reveals the probable mechanisms underlying the antipyretic efficacy of WBH at a metabolic level.

## Supporting Information

S1 File This file contains Figures A to I. (DOCX)

S1 Table Identified differential metabolites selected by OPLS-DA with VIP>2 and discrimination between fever and normal group in plasma and urine. (DOC)

S2 Table Identified differential metabolites selected by OPLS-DA with VIP>2 and discrimination normal, fever, WBH-treated and aspirin-treated group in plasma and urine. (DOC)

S3 Table Data of heatmaps. (XLSX)
