# Supplementary material for: Metabolomics of the Antipyretic Effects of Bubali Cornu (Water Buffalo Horn) in Rats
Source: PLoS One. 2016 Jul 6;11(7):e0158478. doi: 10.1371/journal.pone.0158478 (PMC4934856; doi:10.1371/journal.pone.0158478)
Supplement: S1 File — (DOCX) [file pone.0158478.s001.docx]

**A Fig**. **Typical BPI chromatogram of plasma.** (a) normal rat in positive mode, (b) fever rat in positive mode; (c) normal rat in negative mode, (d) fever rat in negative mode.

**B Fig**. **Typical BPI chromatogram of urine.** (a) normal rat in positive mode, (b) fever rat in positive mode; (c) normal rat in negative mode, (d) fever rat in negative mode.

**C Fig**. PCA model results of plasma between normal group and fever group in positive mode (a) and negative mode (b). S-plot of OPLS-DA model for fever group vs normal group (c, positive mode; d, negative mode).

**D Fig**. PCA model results of urine between normal group and fever group in positive mode (a) and negative mode (b). S-plot of OPLS-DA model for fever group vs normal group (c, positive mode; d, negative mode).

**E Fig**. OPLS-DA model results of plasma among normal group, fever group, WBH treated fever group and Aspirin treated fever group in positive mode (a) and negative mode (b). Loading plot of four groups from OPLS-DA (c, positive mode; d, negative mode).

**F Fig**. OPLS-DA model results of urine among normal group, fever group, WBH treated fever group and Aspirin treated fever group in positive mode (a) and negative mode (b). Loading plot of four groups from OPLS-DA (c, positive mode; d, negative mode).

**G Fig**. **Heat maps from UPLC-MS.** Fingerprinting of endogenous metabolites from the Normal group, Fever group, WBH treated group and Aspirin treated group. Green represents negative values, red represents positive values.

**H Fig**. UPLC-MS/MS chromatograms of plasma sample and standards including glycoursodeoxycholic acid, deoxycholic acid, and prostaglandin E_1_.

**I Fig**. **Summary of pathway analysis of Aspirin with MetPA.** (1) Glycerophospholipid metabolism; (2) Starch and sucrose metabolism; (3) Sphingolipid metabolism; (4) Galactose metabolism; (5) Amino sugar and nucleotide sugar metabolism; (6) Arginine and proline metabolism; (7) Tyrosine metabolism; (8) Glycosylphosphatidylinositol(GPI)-anchor biosynthesis; (9) Purine metabolism; (10) Pyrimidine metabolism; (11) Primary bile acid biosynthesis.


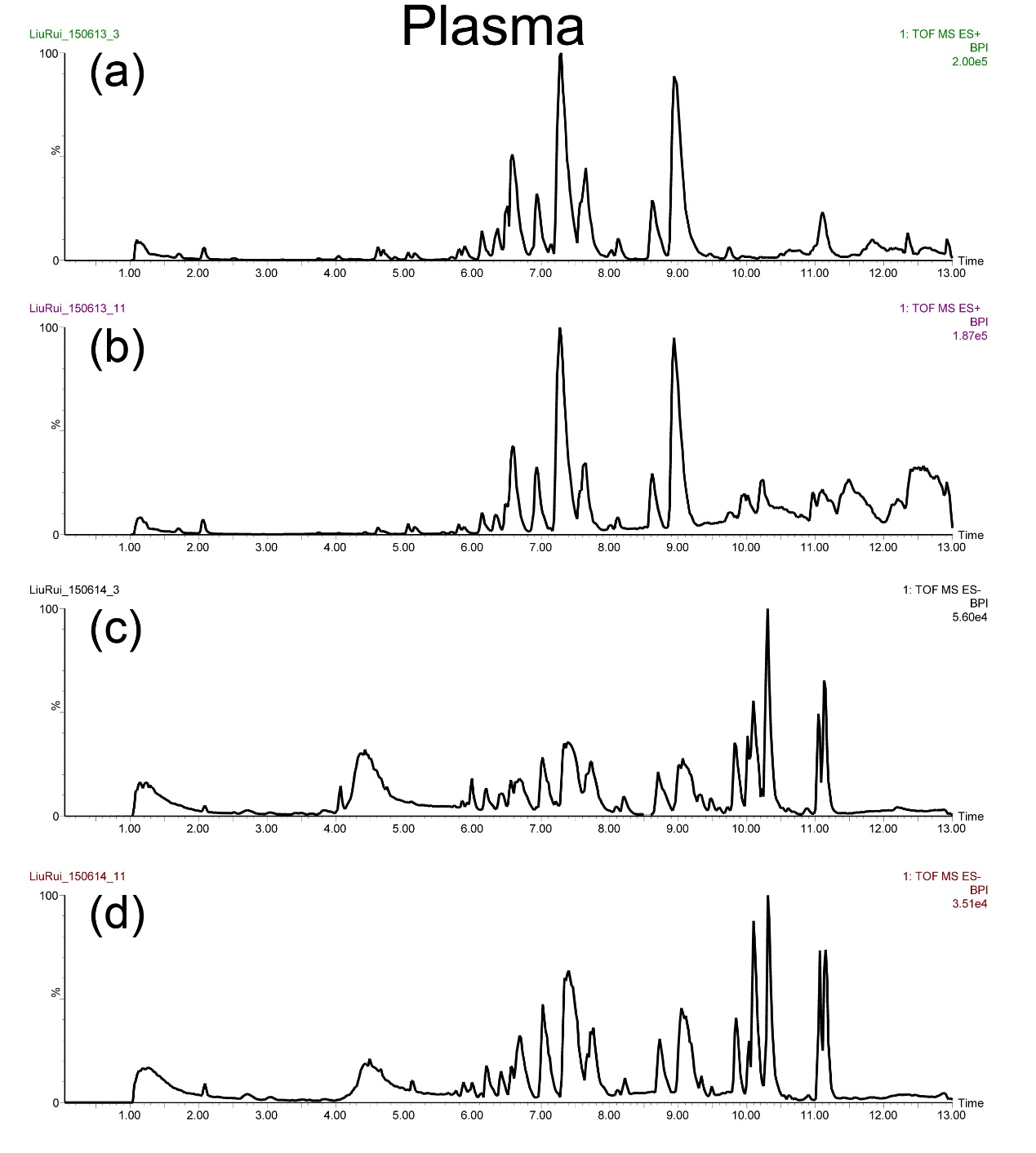


A Fig


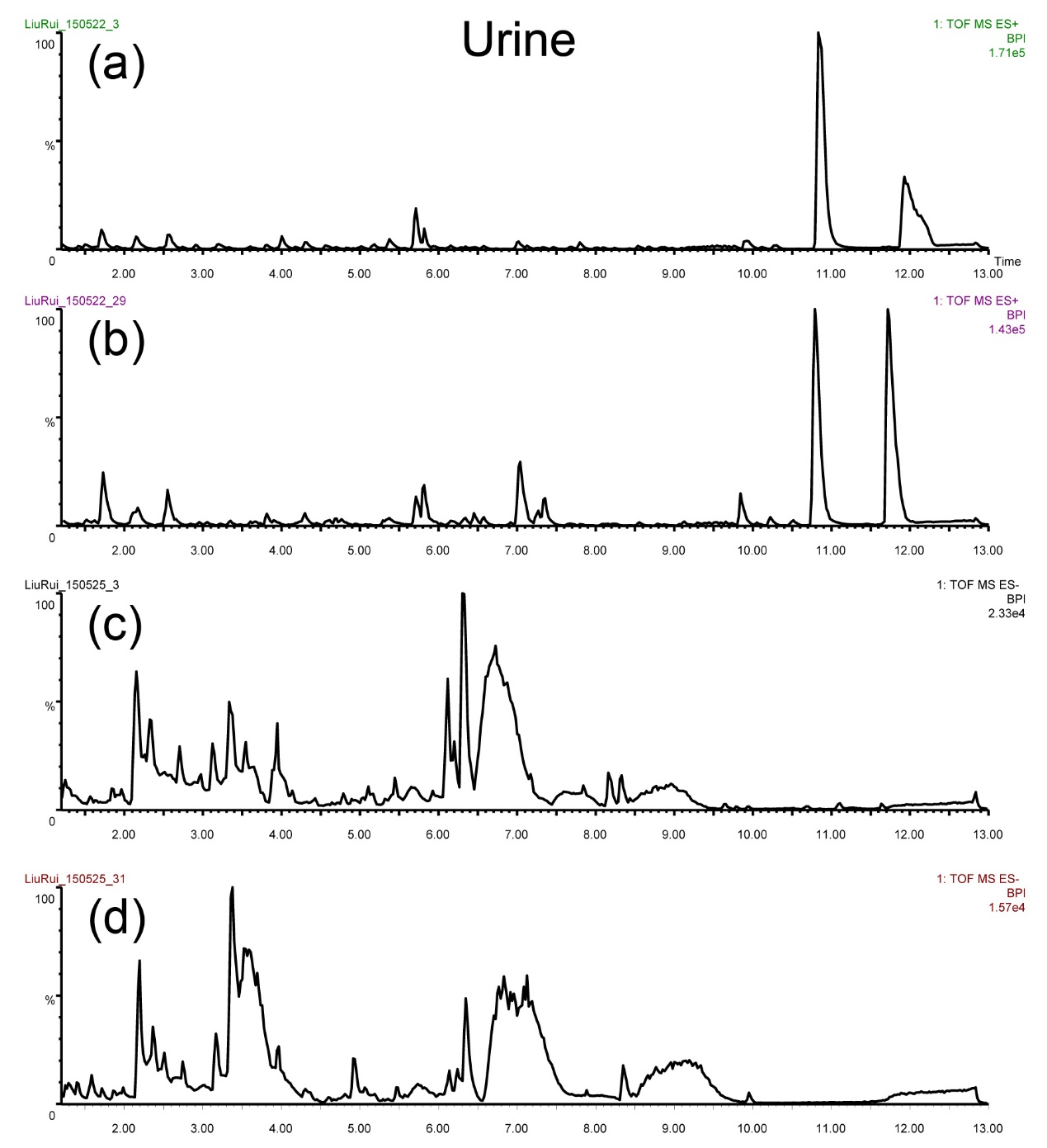


B Fig


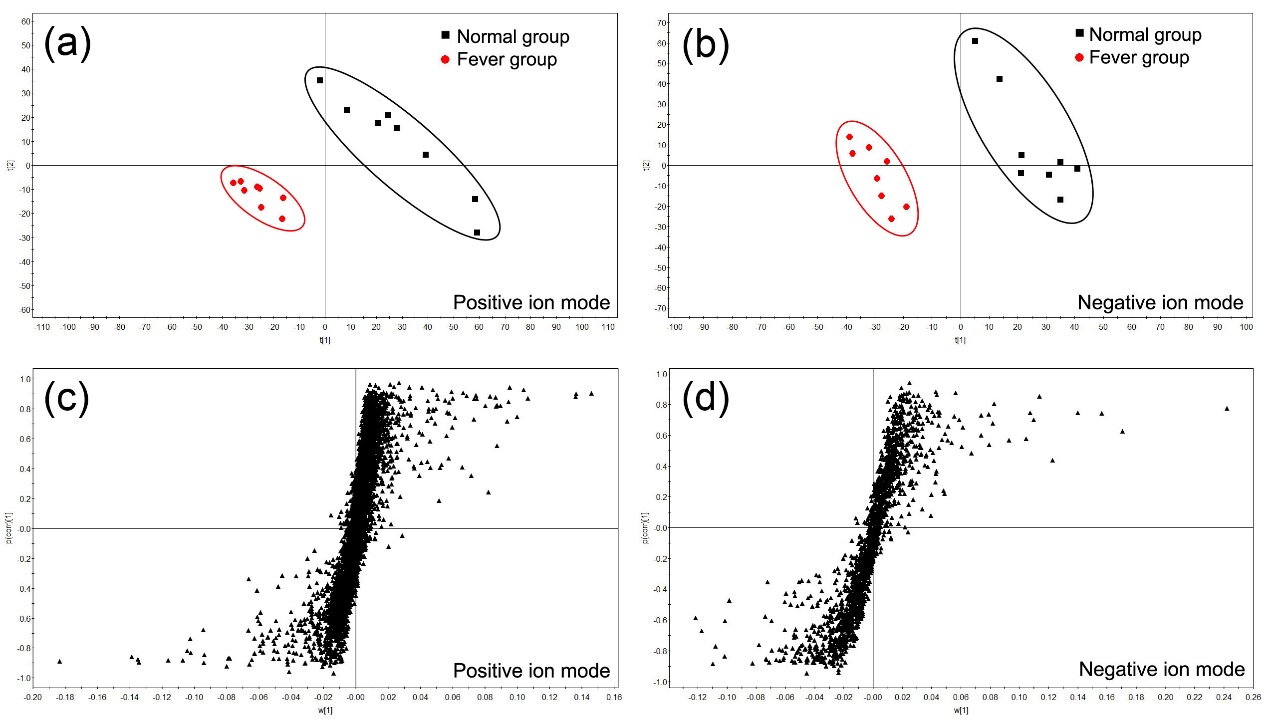


C Fig


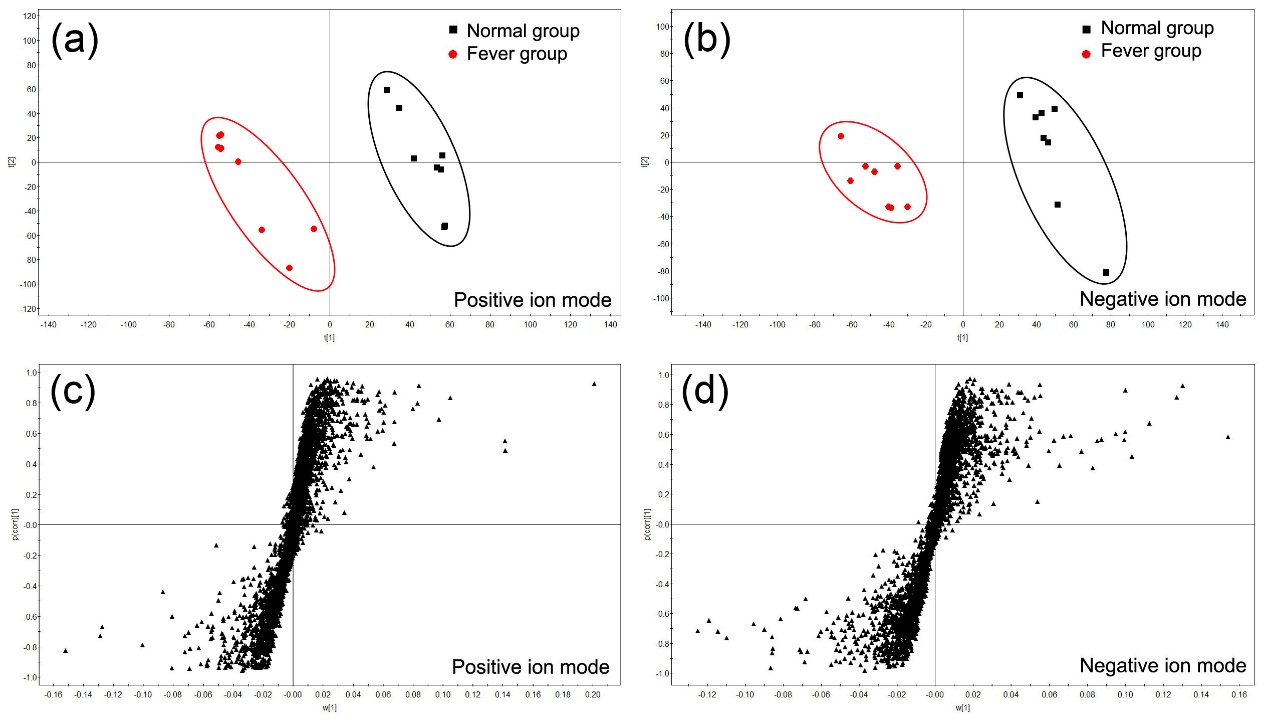


D Fig


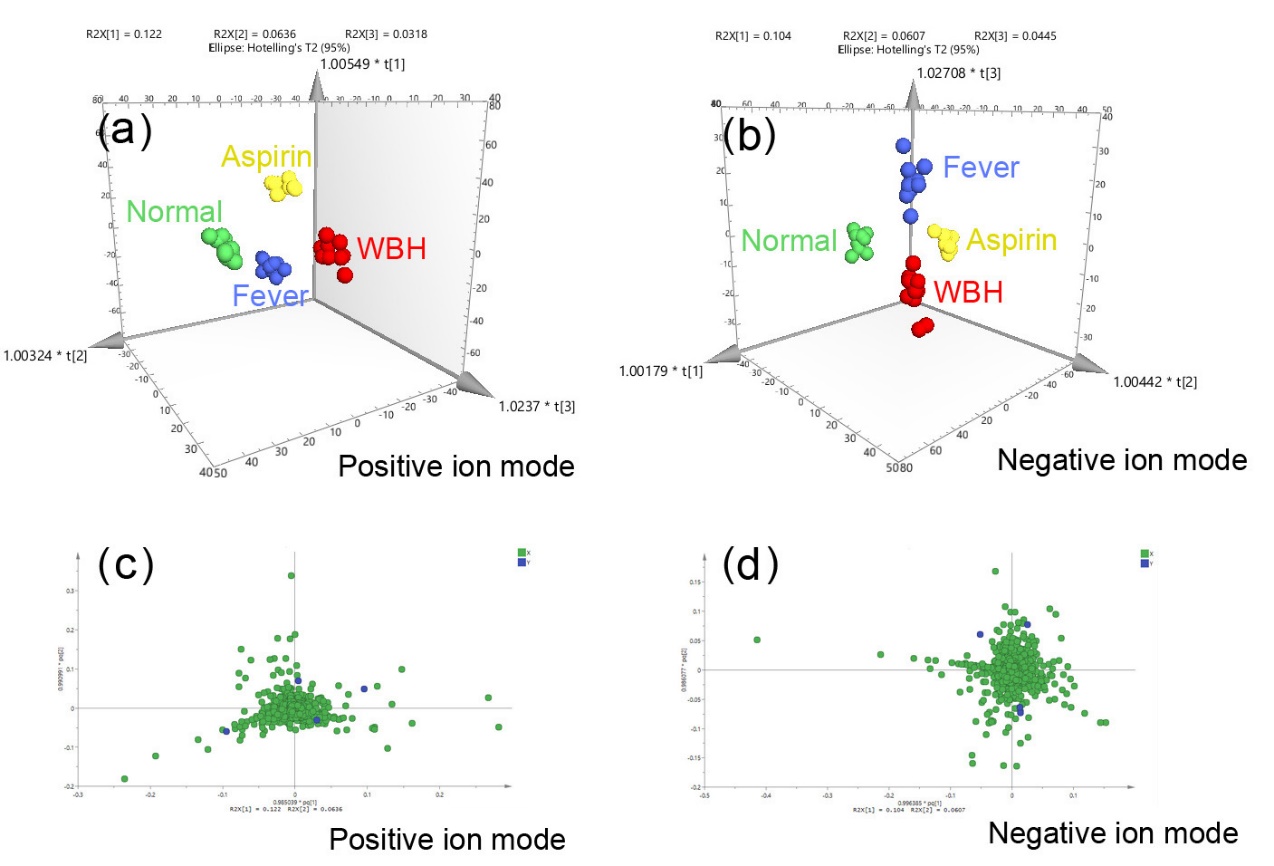


E Fig


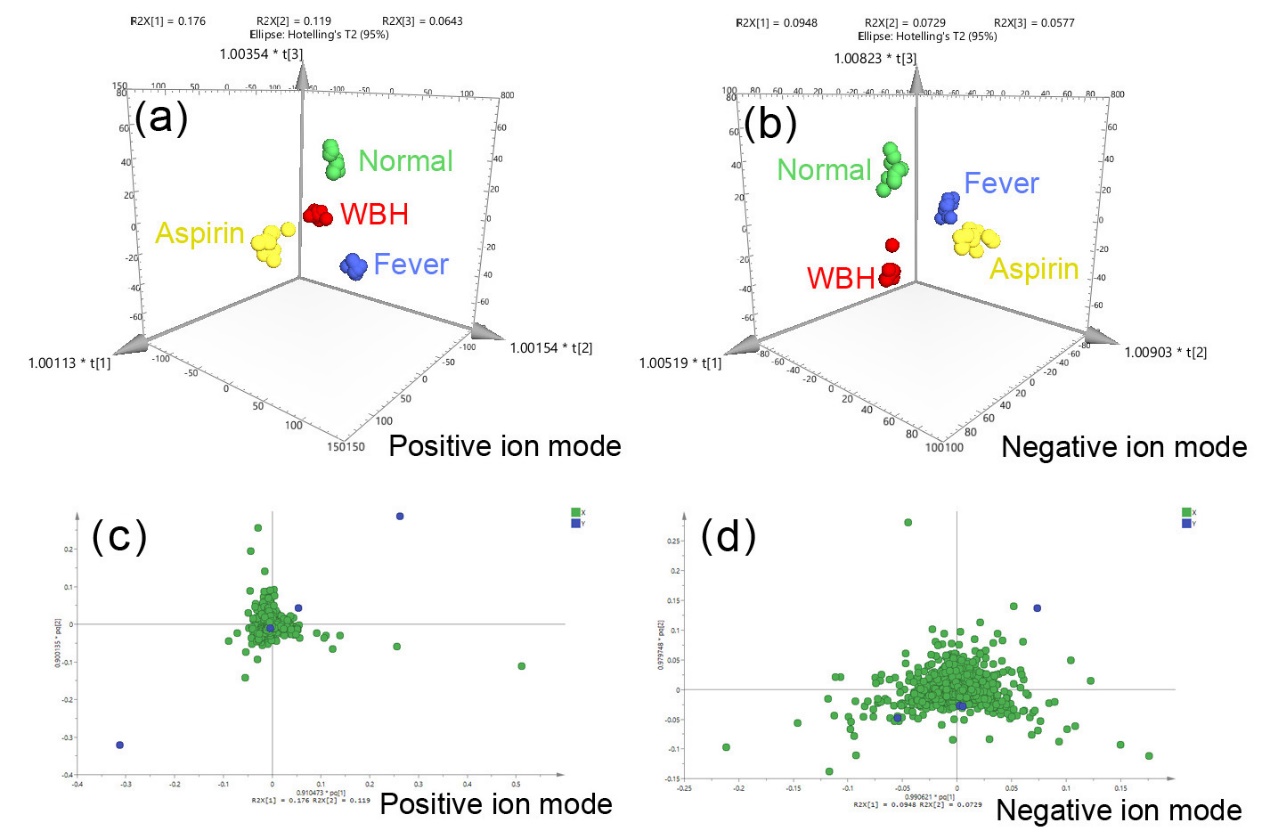


F Fig


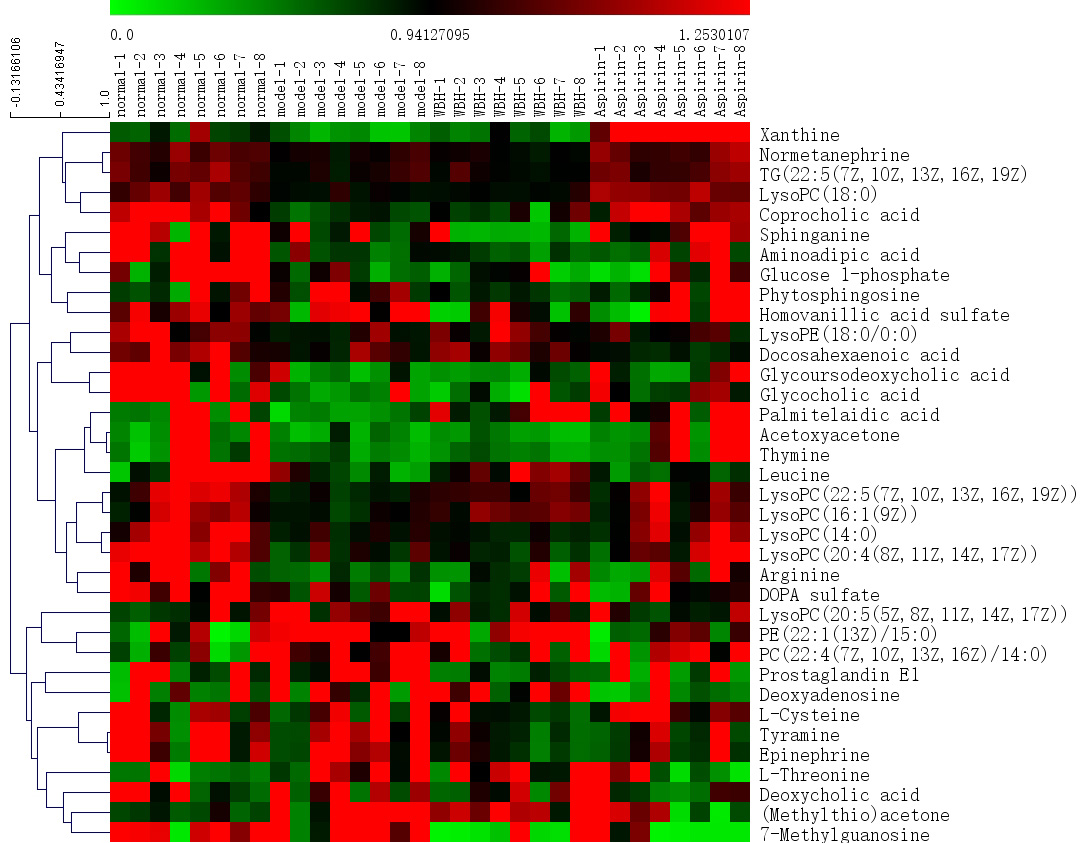


G Fig


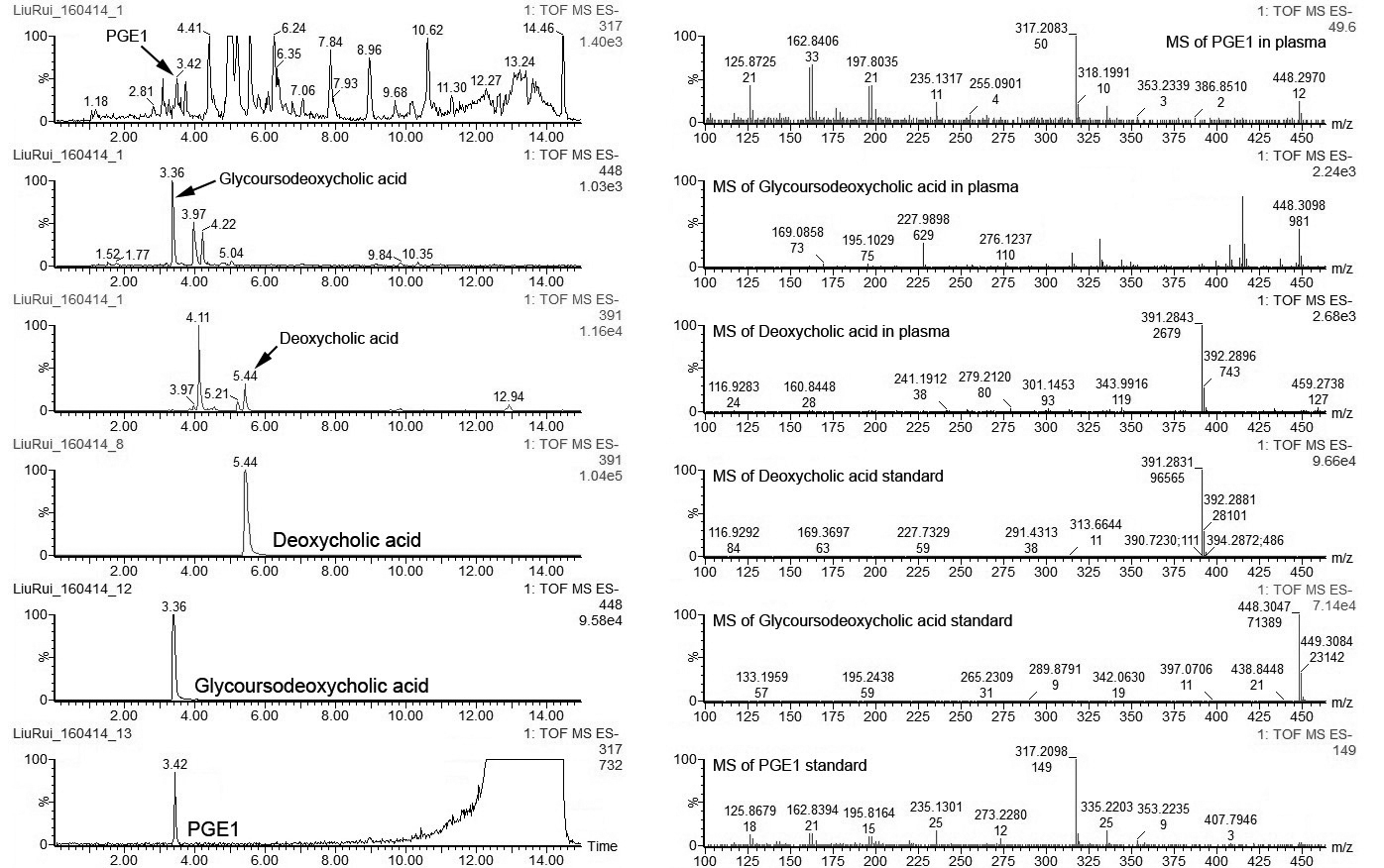
H Fig


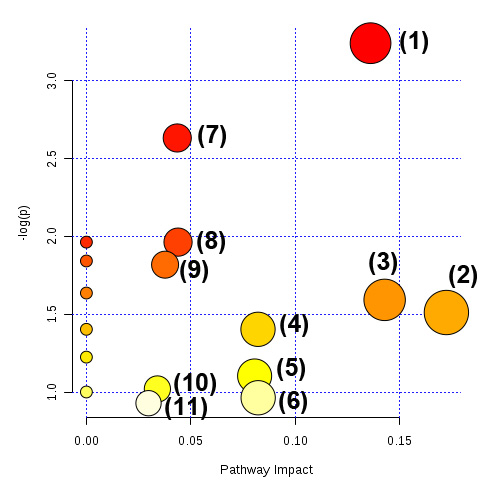


I Fig
