# Supplementary material for: Metabolomics of the Antipyretic Effects of Bubali Cornu (Water Buffalo Horn) in Rats
Source: PLoS One. 2016 Jul 6;11(7):e0158478. doi: 10.1371/journal.pone.0158478 (PMC4934856; doi:10.1371/journal.pone.0158478)
Supplement: S1 Table — (DOC) [file pone.0158478.s002.doc]

Table S1. Identified differential metabolites selected by OPLS-DA with VIP>2 and discrimination between fever and normal group in plasma and urine

| Positive mode | | | | | | | | | |
| --- | --- | --- | --- | --- | --- | --- | --- | --- | --- |
| Plasma | | | | | | | | | |
| No. | t_R_ (min) | VIP | Obsd [M+H]+ | Calcd [M+H]+ | Metabolites | MS/MS | M vs. C | WBH vs. M | Related pathway |
| 1 | 9.74 | 6.260 | 538.3864 | 538.3867 | LysoPE(22:0/0:0) | 520.3739, 496.343, 355.3194, 184.0728, 124.9972, 104.1066 | ↓### | ↑* | Glycerophospholipid metabolism |
| 2 | 6.59 | 8.595 | 544.3390 | 544.3398 | LysoPC(20:4(5Z,8Z,11Z,14Z)) | 520.3382, 502.325, 184.0741, 124.9977, 104.1044 | ↓# | ↑* | Glycerophospholipid metabolism |
| 3 | 7.15 | 4.240 | 546.3528 | 546.3554 | LysoPC(20:3(5Z,8Z,11Z)) | 528.3419, 478.3307, 184.0719, 141.1148, 124.9997 | ↓# | ↓* | Glycerophospholipid metabolism |
| 4 | 9.16 | 4.330 | 550.3875 | 550.3867 | LysoPC(20:1(11Z)) | 524.3598, 506.3555, 184.0748, 166.0635, 124.9983, 104.1055 | ↓## | ↑* | Glycerophospholipid metabolism |
| 5 | 6.50 | 6.181 | 568.3399 | 568.3398 | LysoPC(22:6(4Z,7Z,10Z,13Z,16Z,19Z)) | 482.3195, 464.3141, 184.0727, 124.9986, 104.1057 | ↓# | - | Glycerophospholipid metabolism |
| 6 | 6.14 | 5.062 | 494.3242 | 494.3241 | LysoPC(16:1(9Z)) | 476.3116, 184.0710, 124.9997, 104.1066 | ↓# | - | Glycerophospholipid metabolism |
| Urine | | | | | | | | | |
| 7 | 11.02 | 3.079 | 330.3369 | 330.3003 | Dihydroceramide | 312.3229, 215.1347, 133.0914 | ↑### | ↓** | Sphingolipid metabolism |
| 8 | 8.92 | 2.216 | 231.1433 | 231.1591 | Dodecanedioic acid | 153.1303 | ↑# | ↓** | Unknown |
| 9 | 4.04 | 2.883 | 135.0832 [M+Na]+ | 135.0165 | Uracil | - | ↑# | ↓** | Pyrimidine metabolism |
| 10 | 7.07 | 10.491 | 255.0662 | 255.1452 | Homoanserine | 227.0726, 199.0788, 181.0684 | ↑ | ↑* | Unknown |
| 11 | 8.93 | 3.473 | 153.1301 | 153.0407 | Xanthine | 135.121, 109.1047 | ↑# | ↓** | Purine metabolism |
| 12 | 3.68 | 8.650 | 377.1435  [M+Na]+ | 377.2298 | Prostaglandin E1 | 321.1507, 260.148, 242.1410, 214.1492, 128.0391 | ↑# | ↓** | Arachidonic acid metabolism |
| 13 | 2.66 | 3.912 | 162.0564 | 162.0761 | Aminoadipic acid | 144.046, 116.0517 | ↑ | ↓ | Lysine biosynthesis |
| 14 | 9.08 | 3.995 | 314.2315 | 314.2690 | Palmitoylglycine | 246.2415 | ↑### | ↓*** | Unknown |
| Negative mode | | | | | | | | | |
| Plasma | | | | | | | | | |
| 15 | 4.39 | 3.997 | 333.2040 | 333.2071 | Prostaglandin J2 | 315.1918, 257.1552, 152.9961, 116.9262 | ↑# | ↓* | Arachidonic acid metabolism |
| 16 | 5.00 | 2.381 | 285.1836 | 285.2071 | Hexadecanedioic acid | 257.6601 | ↑# | ↓** | Unknown |
| 17 | 5.65 | 4.291 | 317.2096 | 317.2122 | Leukotriene A4 | 299.2015 | ↑### | ↓** | Arachidonic acid metabolism |
| 18 | 4.78 | 3.173 | 391.2812 | 391.2854 | Deoxycholic acid | 345.2885 | ↓# | ↑ | Secondary bile acid biosynthesis |
| 19 | 4.92 | 2.490 | 448.3038 | 448.3068 | Glycoursodeoxycholic acid | - | ↓## | - | Secondary bile acid biosynthesis |
| 20 | 4.08 | 6.157 | 464.2993 | 464.3018 | Glycocholic acid | 448.3044 | ↓## | ↓ | Primary bile acid biosynthesis |
| 21 | 7.42 | 4.832 | 480.3094 | 480.3096 | LysoPE(18:0/0:0) | 452.2744, 368.6375, 281.9089, 203.3595, 134.254 | ↑# | ↑* | Glycerophospholipid metabolism |
| 22 | 4.24 | 3.062 | 493.2425 | 493.2378 | Leukotriene D5 | 317.2094, 300.6625 | ↑### | ↓** | Arachidonic acid metabolism |
| 23 | 5.96 | 2.476 | 526.2922 | 526.2939 | LysoPE(0:0/22:5(7Z,10Z,13Z,16Z,19Z)) | 391.2813, 345.2623, 301.2119 | ↓# | ↑ | Glycerophospholipid metabolism |
| 24 | 9.12 | 3.990 | 568.3618 | 568.3409 | LysoPC(22:5(4Z,7Z,10Z,13Z,16Z)) | 508.3406, 283.2640, 164.2178 | ↑# | ↑* | Glycerophospholipid metabolism |
| Urine | | | | | | | | | |
| 25 | 5.79 | 3.184 | 248.0941 | 248.0234 | Norepinephrine sulfate | 236.0938, 197.0861, 153.0925, 116.0516 | ↑# | ↑ | Tyrosine metabolism |
| 26 | 7.39 | 3.190 | 250.1096 | 250.0946 | Deoxyadenosine | 198.1103, 142.0566, 100.0088 | ↑# | ↑ | Purine metabolism |
| 27 | 7.24 | 7.058 | 253.0493 | 253.1306 | Homoanserine | - | ↑ | ↑* | Unknown |
| 28 | 7.17 | 3.892 | 155.1063 | 155.1078 | 4-Hydroxynonenal | - | ↑# | - | Unknown |
| 29 | 3.08 | 2.512 | 273.0061 | 273.0017 | D-Glucuronic acid 1-phosphate | 193.0476, 133.0305 | ↓# | - | Ascorbate and aldarate metabolism |
| 30 | 5.06 | 3.726 | 172.0978  [M+Cl]^－^ | 172.0535 | Tyramine | 119.0499, 107.0505 | ↑# | - | Tyrosine metabolism |
| 31 | 2.80 | 3.831 | 172.9903  [M+Cl]^－^ | 173.0123 | Urocanic acid | 137.0603 | ↑# | ↓ | Histidine metabolism |
| 32 | 3.05 | 5.938 | 275.0208  [M+Cl]^－^ | 274.9933 | Cystine | - | ↓# | ↑* | Cysteine and methionine metabolism |
| 33 | 4.79 | 6.641 | 283.0805 | 283.0684 | Xanthosine | 231.0767 | ↑## | ↓* | Purine metabolism |
| 34 | 4.58 | 5.298 | 187.0060 | 187.0724 | N-Acetylglutamine | - | ↑# | ↑ | Arginine biosynthesis |
| 35 | 2.40 | 5.310 | 188.9848  [M+Cl]^－^ | 188.9960 | 2-Pyrocatechuic acid | 109.0297 | ↓# | ↑ | Benzoate degradation |
| 36 | 3.99 | 7.193 | 295.1281 | 295.0936 | Aspartyl-Tyrosine | 256.0602, 192.0654 | ↓# | ↑ | Unknown |
| 37 | 2.58 | 5.524 | 308.1143 | 308.1014 | Tyrosyl-Glutamine | 280.0851, 188.9859, 158.082 | ↓# | ↑* | Unknown |
| 38 | 3.37 | 6.332 | 212.0010 | 211.9966 | L-Aspartyl-4-phosphate | - | ↑# | ↑ | Cysteine and methionine metabolism |
| 39 | 8.40 | 4.055 | 329.1581  [M+Cl]^－^ | 329.1889 | 17-Hydroxyprogesterone | 158.1195 | ↑# | ↓* | Steroid hormone biosynthesis |
