# Supplementary material for: Metabolomics of the Antipyretic Effects of Bubali Cornu (Water Buffalo Horn) in Rats
Source: PLoS One. 2016 Jul 6;11(7):e0158478. doi: 10.1371/journal.pone.0158478 (PMC4934856; doi:10.1371/journal.pone.0158478)
Supplement: S2 Table — (DOC) [file pone.0158478.s003.doc]

Table S2. Identified differential metabolites selected by OPLS-DA with VIP>2 and discrimination normal, fever, WBH-treated and aspirin-treated group in plasma and urine

| Positive mode | | | | | | | | | |  |
| --- | --- | --- | --- | --- | --- | --- | --- | --- | --- | --- |
| Plasma | | | | | | | | | |  |
| No. | t_R_ (min) | VIP | Obsd [M+H]+ | Calcd [M+H]+ | Metabolites | M vs. C | APC vs. M | WBH vs. M | Related pathway | |
| 1 | 8.41 | 13.04 | 524.3688 | 524.3711 | LysoPC(18:0) | ↓# | ↑*** | - | Glycerophospholipid metabolism | |
| 2 | 6.79 | 3.98 | 184.0740 | 184.0968 | Normetanephrine | ↓## | ↑* | - | Tyrosine Metabolism | |
| 3 | 5.86 | 2.36 | 542.3250 | 542.3241 | LysoPC(20:5(5Z,8Z,11Z,14Z,17Z)) | ↑# | ↓* | ↓** | Glycerophospholipid metabolism | |
| 4 | 5.41 | 2.52 | 255.2099 | 255.2318 | Palmitelaidic acid | ↓# | ↑*** | ↑*** | Unknown | |
| 5 | 7.04 | 8.42 | 991.6779 | 991.7749 | TG(22:5(7Z,10Z,13Z,16Z,19Z) | ↓# | ↑** | - | Glycerophospholipid metabolism | |
| 6 | 10.20 | 3.81 | 760.5889 | 760.5851 | PE(22:1(13Z)/15:0) | ↑## | ↓*** | - | Glycerophospholipid metabolism | |
| 7 | 7.37 | 2.28 | 482.3615 | 482.3241 | LysoPE(18:0/0:0) | ↓## | ↑* | ↑* | Glycerophospholipid metabolism | |
| 8 | 10.23 | 2.59 | 782.5641 | 782.5694 | PC(22:4(7Z,10Z,13Z,16Z)/14:0) | ↑## | - | ↓* | Glycerophospholipid metabolism | |
| Urine | | | | | | | | | |  |
| 9 | 3.33 | 2.22 | 105.0353 | 105.0369 | (Methylthio)acetone | ↑## | ↓* | - | Unknown | |
| 10 | 4.95 | 2.12 | 120.0839 | 120.0655 | L-Threonine | ↑## | ↓* | - | Glycine, serine and threonine metabolism | |
| 11 | 10.48 | 2.48 | 302.3062 | 302.3054 | Sphinganine | ↓# | ↓** | ↑* | Sphingolipid metabolism | |
| 12 | 11.73 | 2.45 | 139.0064  [M+Na]+ | 139.0366 | Acetoxyacetone | ↓ | ↑* | - | Unknown | |
| 13 | 2.68 | 2.32 | 144.0470  [M+Na]+ | 144.0090 | L-Cysteine | ↓# | - | ↑* | Cysteine and methionine metabolism | |
| 14 | 9.83 | 2.65 | 318.2960 | 318.3003 | Phytosphingosine | ↑# | - | ↓** | Sphingolipid metabolism | |
| 15 | 11.44 | 2.07 | 149.0268  [M+Na]+ | 149.0321 | Thymine | ↓# | ↑* | - | Pyrimidine metabolism | |
| 16 | 2.25 | 2.28 | 160.0417  [M+Na]+ | 160.0733 | Tyramine | ↑ | ↓* | ↓** | Tyrosine metabolism | |
| 17 | 3.68 | 4.19 | 377.1439  [M+Na]+ | 377.2298 | Prostaglandin E1 | ↑# | ↓** | ↓* | Arachidonic acid metabolism | |
| Negative mode | | | | | | | | | |  |
| Plasma | | | | | | | | | |  |
| 18 | 7.62 | 2.12 | 604.3374  [M+Cl]^－^ | 604.3175 | LysoPC(22:5(7Z,10Z,13Z,16Z,19Z)) | ↓### | ↑** | ↑*** | Glycerophospholipid metabolism | |
| 19 | 2.31 | 3.86 | 187.0057  [M+Cl]^－^ | 187.0028 | Xanthine | ↓## | ↑*** | ↑** | Caffeine metabolism | |
| 20 | 9.11 | 5.13 | 327.2319 | 327.2329 | Docosahexaenoic acid | ↓# | ↓* | ↑* | Alpha Linolenic Acid and Linoleic Acid Metabolism | |
| 21 | 4.57 | 3.53 | 391.2833 | 391.2854 | Deoxycholic acid | ↓# | - | ↑ | Secondary bile acid biosynthesis | |
| 22 | 4.73 | 2.36 | 448.3063 | 448.3068 | Glycoursodeoxycholic acid | ↓## | ↑* | - | Secondary bile acid biosynthesis | |
| 23 | 3.77 | 3.28 | 464.3005 | 464.3018 | Glycocholic acid | ↓## | ↑** | - | Primary bile acid biosynthesis | |
| 24 | 7.35 | 2.24 | 466.3287 | 466.2939 | LysoPC(14:0) | ↓## | ↑* | - | Glycerophospholipid metabolism | |
| 25 | 10.32 | 2.77 | 485.2817  [M+Cl]^－^ | 485.3039 | Coprocholic acid | ↓## | ↑** | - | Unknown | |
| 26 | 7.62 | 2.19 | 492.3450 | 492.3096 | LysoPC(16:1(9Z)) | ↓### | ↑* | ↑*** | Glycerophospholipid metabolism | |
| 27 | 7.36 | 2.09 | 578.3227 | 578.3019 | LysoPC(20:4(8Z,11Z,14Z,17Z)) | ↓## | ↑* | - | Glycerophospholipid metabolism | |
| Urine | | | | | | | | | |  |
| 28 | 4.50 | 3.23 | 130.0867 | 130.0874 | Leucine | ↓## | - | ↑*** | Valine, leucine and isoleucine biosynthesis | |
| 29 | 2.39 | 3.07 | 160.0391 | 160.0615 | Aminoadipic acid | ↓## | ↑* | - | Lysine biosynthesis | |
| 30 | 5.04 | 3.10 | 173.0821 | 173.1044 | Arginine | ↓# | ↑* | - | Arginine and proline metabolism | |
| 31 | 7.39 | 3.36 | 250.1094 | 250.0946 | Deoxyadenosine | ↑ | ↑ | ↓* | Purine metabolism | |
| 32 | 4.48 | 2.74 | 259.1178 | 259.0224 | Glucose 1-phosphate | ↓## | ↑* | - | Amino sugar and nucleotide sugar metabolism | |
| 33 | 2.26 | 4.28 | 261.0048 | 261.0074 | Homovanillic acid sulfate | ↑# | - | ↓** | Tyrosine metabolism | |
| 34 | 2.50 | 2.50 | 276.0527 | 276.0183 | DOPA sulfate | ↓### | - | ↑* | Unknown | |
| 35 | 6.49 | 4.33 | 297.0949 | 297.1079 | 7-Methylguanosine | ↑# | ↓*** | ↓*** | Unknown | |
